# Progressive mitochondrial dysfunction in cerebellar synaptosomes of cystatin B-deficient mice

**DOI:** 10.3389/fnmol.2023.1175851

**Published:** 2023-05-12

**Authors:** Katarin Gorski, Christopher B. Jackson, Tuula A. Nyman, Veronika Rezov, Brendan J. Battersby, Anna-Elina Lehesjoki

**Affiliations:** ^1^Folkhälsan Research Center, Helsinki, Finland; ^2^Medicum, Faculty of Medicine, University of Helsinki, Helsinki, Finland; ^3^Department of Biochemistry and Developmental Biology, Medicum, Faculty of Medicine, University of Helsinki, Helsinki, Finland; ^4^Department of Immunology, Oslo University Hospital, University of Oslo, Oslo, Norway; ^5^Institute of Biotechnology, University of Helsinki, Helsinki, Finland

**Keywords:** mitochondria, myoclonus, neurodegeneration, OXPHOS, synaptosome, respiration, proteomics

## Abstract

The involvement of mitochondrial dysfunction in cystatin B (CSTB) deficiency has been suggested, but its role in the onset of neurodegeneration, myoclonus, and ataxia in the CSTB-deficient mouse model (*Cstb^−/−^*) is yet unknown. CSTB is an inhibitor of lysosomal and nuclear cysteine cathepsins. In humans, partial loss-of-function mutations cause the progressive myoclonus epilepsy neurodegenerative disorder, EPM1. Here we applied proteome analysis and respirometry on cerebellar synaptosomes from early symptomatic (*Cstb^−/−^*) mice to identify the molecular mechanisms involved in the onset of CSTB-deficiency associated neural pathogenesis. Proteome analysis showed that CSTB deficiency is associated with differential expression of mitochondrial and synaptic proteins, and respirometry revealed a progressive impairment in mitochondrial function coinciding with the onset of myoclonus and neurodegeneration in (*Cstb^−/−^*) mice. This mitochondrial dysfunction was not associated with alterations in mitochondrial DNA copy number or membrane ultrastructure. Collectively, our results show that CSTB deficiency generates a defect in synaptic mitochondrial bioenergetics that coincides with the onset and progression of the clinical phenotypes, and thus is likely a contributor to the pathogenesis of EPM1.

## 1. Introduction

Progressive myoclonus epilepsy EPM1 (Unverricht-Lundborg disease; OMIM 254800), caused by biallelic partial loss-of-function mutations in the cystatin B (*CSTB*) gene (Pennacchio et al., 1996; Joensuu et al., 2008), is a neurodegenerative disorder manifesting with minor or no cognitive decline (Koskiniemi et al., 1974; Kälviäinen et al., 2008). Patients develop severely disabling and treatment-resistant myoclonus and tonic–clonic epileptic seizures between 6 and 16 years of age, followed by ataxia, incoordination and dysarthria (Koskiniemi et al., 1974; Kälviäinen et al., 2008). Magnetic resonance imaging of EPM1 patient brains show widespread degenerative changes in both white and grey matter (Koskenkorva et al., 2009, 2012; Manninen et al., 2013), and MRI-navigated transcranial magnetic stimulation analyses altered cortical responses (Danner et al., 2009; Julkunen et al., 2013). Postmortem analyses have shown widespread atrophy, neuronal loss, and gliosis in both cerebrum and cerebellum (Haltia et al., 1969; Koskiniemi et al., 1974; Eldridge et al., 1983; Cohen et al., 2011).

Most EPM1 patients are homozygous for a 12-nucleotide repeat expansion mutation in the promoter region of *CSTB*, reducing *CSTB* mRNA and protein expression to less than 10% of that in controls (Joensuu et al., 2007). Patients that are compound heterozygous for the repeat expansion and a truncating null mutation have a more severe phenotype with an earlier onset and poorer cognitive performance (Koskenkorva et al., 2011; Canafoglia et al., 2012). In contrast, patients homozygous for two null mutations manifest a severe neonatal-onset progressive encephalopathy that is clinically distinct from EPM1 (Mancini et al., 2016; O’Brien et al., 2017). These genotype–phenotype correlations suggest that CSTB is essential for normal brain development and for maintaining neuronal integrity in mature brain. The CSTB deficient knockout mouse (*Cstb^−/−^*) is a model for EPM1. It recapitulates the key clinical features and pathological changes of the disorder: myoclonus, progressive ataxia, and grey and white matter degeneration (Pennacchio et al., 1998; Tegelberg et al., 2012; Manninen et al., 2013, 2014).

Cystatin B is a ubiquitously expressed inhibitor of cysteine proteases of the cathepsin family (Green et al., 1984; Čeru et al., 2010) showing both cytoplasmic and nuclear localization. In the cytoplasm, CSTB partially co-localizes with lysosomal markers (Alakurtti et al., 2005) and is thought to prevent inappropriate proteolytic activity and redistribution of cysteine cathepsins in the cytosol (Boya and Kroemer, 2008). In line with its lysosomal association, CSTB function has been linked to protecting neurons from oxidative damage through an oxidative stress-responsive cystatin B-cathepsin B signaling pathway (Lehtinen et al., 2009). In the nucleus, CSTB interacts with histones and cathepsin L, which affects cell cycle regulation and proteolytic cleavage of the histone H3 tail (Čeru et al., 2010; Daura et al., 2021). Downstream effects of CSTB function are implicated in several cellular and biological processes, including inflammation (Tegelberg et al., 2012; Maher et al., 2014; Okuneva et al., 2016), apoptosis (Pennacchio et al., 1998), neurogenesis (Di Matteo et al., 2020; Daura et al., 2021), and synapse physiology (Joensuu et al., 2014; Penna et al., 2019; Gorski et al., 2020). CSTB deficiency induces widespread physiological and pathological changes in the mouse brain, which are most pronounced in the cerebellum. This includes progressive loss of cerebellar granule cells from 1 month of age onwards (Pennacchio et al., 1998) and a decrease in the cerebellar volume by 50% at 6 months of age (Tegelberg et al., 2012). Other changes include altered GABAergic signaling (Joensuu et al., 2014), glial activation (Tegelberg et al., 2012) and inflammation (Okuneva et al., 2016). All of these events precede neuronal death.

To gain insight into the molecular defects associated with synaptic function in *Cstb^−/−^* mice, we previously performed a quantitative proteomics study of cerebellar synaptosomes isolated from presymptomatic two-week old mice (Gorski et al., 2020). We found that one third of the cerebellar synaptosomal proteins that differed in abundance belong to the mitochondrial proteome, primarily those involved with oxidative phosphorylation (OXPHOS). These data imply that mitochondrial dysfunction is associated with the early pathogenesis of altered synaptic function in CSTB deficiency. In line with these findings, neural progenitor cells from *Cstb^−/−^* mice had altered mRNA expression levels of nuclear-encoded OXPHOS genes and impaired mitochondrial respiration upon neural stem cell differentiation (Daura et al., 2021).

In the present study, we investigated cerebellar synaptosomes from symptomatic *Cstb^−/−^* mice early in the phenotype onset. Our results show that changes in the mitochondrial proteome and respiration are linked to the early onset of myoclonus and neurodegeneration. Collectively, our study expands the current understanding of mitochondrial involvement in the early neuronal pathology in CSTB deficiency.

## 2. Materials and methods

### 2.1. Ethics statement

The Animal Ethics Committee of the State Provincial Office of Southern Finland approved all animal research protocols (decisions ESAVI/10765/2015 and ESAVI/471/2019).

### 2.2. Mice

*Cstb^−/−^* mice were derived from The Jackson Laboratory (Bar Harbor, ME; 129-Cstb^tm1Rm^/SvJ; stock #003486) (Pennacchio et al., 1998). Wild type mice of same age and background were used as controls. Heterozygous *Cstb^+/−^* males were backcrossed with inbred wild type females to expand the colony from heterozygous littermates and to maintain the *Cstb^−/−^* mouse line. The genetic background of the mouse colony was refreshed annually with wild type females, and F1-F3 generations were used for experimental procedures. Mice were sacrificed by carbon dioxide euthanasia, followed by cervical dislocation.

### 2.3. Synaptosome isolation

**Figure 1:**
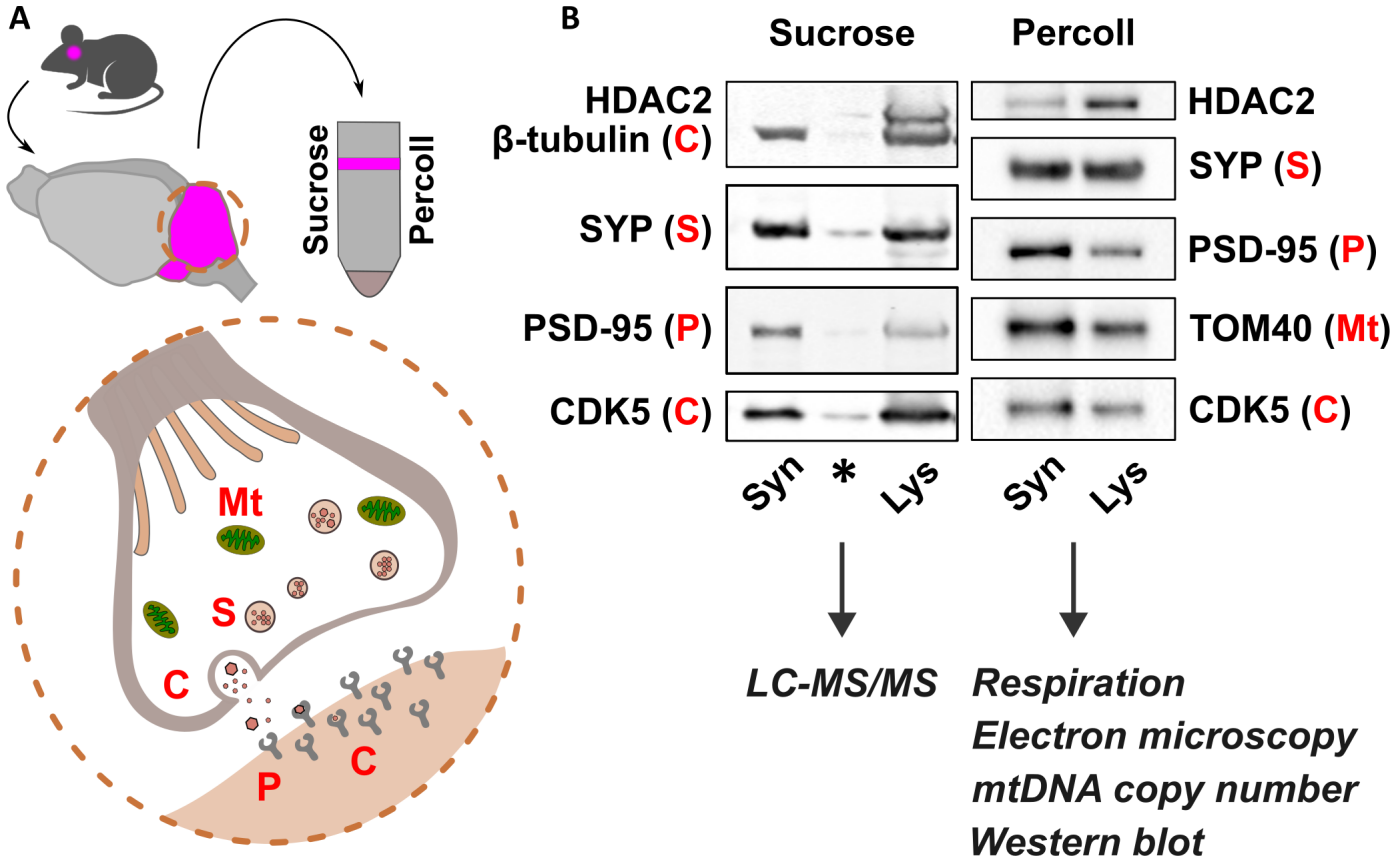
Synaptosomal fractionation and characterization. **(A)** Schematic presentation of synaptosome isolation from mouse cerebella (pink) using sucrose and Percoll. Synaptosome preparations consist of functional pre- and postsynaptic terminals containing organelles, synaptic vesicles, and receptors for neurotransmitters. **(B)** Immunoblot detection of pre- and postsynaptic proteins Synaptophysin (SYP) and Post-synaptic density 95 (PSD-95), respectively, and depletion of Histone deacetylase 2 (HDAC2) show synaptic protein enrichment in synaptosome preparations (Syn) compared to cerebellar lysate (Lys). Mitochondrial abundance is shown in Percoll-fractions by detection of Mitochondrial import receptor subunit TOM40. Cytosolic proteins are represented by β-tubulin and cyclin dependent kinase 5 (CDK5). Sucrose and Percoll-isolated fractions were used for mass spectrometry, and analyses of synaptic mitochondria, respectively. Mt., mitochondria; S, SYP; C, cytoplasm; P, PSD-95. Asterisk indicates carry-over signal.

For proteomics analysis, cerebellar synaptosomes from P30 *Cstb^−/−^* and wild type mice were isolated using a sucrose-based separation protocol, as previously described (Gorski et al., 2020). For analyses of high-resolution respirometry, mitochondrial DNA (mtDNA) copy number, immunoblot, and electron microscopy, cerebellar synaptosomes from P30 and P45 *Cstb^−/−^* and wild type mice were isolated using a Percoll-based separation protocol, modified from protocols by Dunkley et al. (2008) and Tenreiro et al. (2017). Briefly, cerebella were dissected and rinsed three times in ice-cold homogenization buffer (H1; 0.32 M sucrose, 1 mM EDTA, 5 mM Tris, pH 7.4), followed by homogenization with 10 even strokes in ice-cold H1 using a glass-Teflon homogenizer. The homogenizer was rinsed with an equal volume of H1, and the combined homogenate was centrifuged at 1000 × *g* for 10 min at +4°C. The resulting supernatant was further centrifuged at 14000 × *g* for 20 min at +4°C. The resulting pellet was resuspended in 45% Percoll (Cytiva, MA, United States) (v/v; Percoll:H1), and centrifuged at 14000 × *g* for 2 min at +4°C. The synaptosomal-enriched fraction on the top layer was washed with four volumes H1 and pelleted twice at 14000 × *g* for 2 min at +4°C with an additional H1 wash in between. Synaptosomal purity and protein enrichment were analyzed by immunoblotting (Figure 1).

### 2.4. Proteomics

#### 2.4.1. Sample preparation and LC–MS/MS analysis

Lipids were removed from synaptosome samples (*n* = 5 + 5 mice/genotype) as previously described (Gorski et al., 2020). Briefly, samples were incubated overnight at −20°C in five volumes ice-cold (−20°C) acetone and centrifuged twice at 1000 × *g* for 10 min at +4°C with an additional acetone wash in between. Pellets were air dried for 5 min and resuspended in freshly prepared 6.0 M urea/25 mM ammonium bicarbonate. Protein concentrations (μg/μl) were determined spectrophotometrically using the BCA protein assay kit (Pierce, Thermo Fisher Scientific) according to the manufacturer’s instructions.

For proteome analysis, 5 μg of each sample was reduced and alkylated using dithiothreitol (DTT) and iodoacetamide (IAA), and the urea concentration was diluted to 1 M, followed by overnight digestion with trypsin (Promega Corporation, WI, USA) at +37°C. Peptides were desalted and concentrated by the STAGE-TIP method using a C18 resin disk (3 M Empore). Samples were eluted with 0.1% formic acid/60% acetonitrile, dried, and solubilized in 7 μl 0.1% formic acid prior to mass spectrometry analysis. Each peptide mixture was analyzed using an EASY-nLC system coupled to the QExactive Plus mass spectrometer (ThermoElectron, Bremen, Germany) equipped with the EASY Spray PepMap®RSLC column (C18, 2 μl, 100 Å, 75 μm x 25 cm) using a 120 min LC separation gradient.

#### 2.4.2. Protein identification, label-free quantification and bioinformatic analyses

The resulting MS raw files were submitted to the MaxQuant software (Cox and Mann, 2008) version 1.6.2.10 for protein identification and label-free quantification. Carbamidomethyl (C) was set as a fixed modification, and acetyl (protein N-term), carbamyl (N-term) and oxidation (M) were set as variable modifications. First search peptide tolerance of 20 ppm and main search error 4.5 ppm were used. Trypsin without proline restriction enzyme option was used with two allowed miscleavages. The minimal unique + razor peptides number was set to 1, and the allowed false discovery rate (FDR) was 0.01 (1%) for peptide and protein identification. Label-free quantitation (LFQ) was employed with default settings. The Uniprot database with ‘mouse’ entries (January 2019) was used for the database searches.

The mass spectrometry proteomics data have been deposited to the ProteomeXchange Consortium^1^ *via* the PRIDE partner repository (Vizcaíno et al., 2013) with the dataset identifier PXD040382.

#### 2.4.3. Data processing and analysis

The LFQ values were log10 -transformed, filtered to include only proteins identified and quantified in at least three out of five replicates in at least one experimental group, and missing values were imputed with default settings. Based on Principal component analysis, one wild type sample was removed as an outlier before statistical testing. To find statistically significant differences between the two groups (*Cstb^−/−^* vs. wild type), *t*-test was performed using permutation-based FDR ≤ 0.05 as cut-off.

Downstream analyses were conducted to proteins with a q-value <0.05 using the following softwares and statistical tests: PANTHER (version 17.0, released 2022-22-02) Overrepresentation Test (Released 20,220,712) (Mi et al., 2013) using the Gene Ontology (GO) enrichment analysis tool (Ashburner et al., 2000; Gene Ontology Consortium, 2021) and Reactome pathway analysis (Wu and Haw, 2017) version 65 (released 2021-10-01), using Fisher’s exact test followed by Benjamini–Hochberg correction (FDR) for multiple testing, considering FDR <0.01 statistically significant; DAVID functional annotation clustering version 2021 (Huang et al., 2009a,b), using medium classification stringency, and EASE scoring followed by Benjamini–Hochberg correction (FDR) for multiple testing, considering FDR <0.05 statistically significant, and Heatmapper (Babicki et al., 2016), using average linkage clustering. Mitochondrial proteins and their functions were retrieved using MitoCarta3.0 (Rath et al., 2021). The software Inkscape^2^ was used for visualization of the data.

### 2.5. High-resolution respirometry

Mitochondrial oxygen consumption rates were measured from freshly isolated synaptosome fractions of P30 and P45 *Cstb^−/−^* and wild type mice (*n* = 7 + 7/age group, males and females) using a high-resolution oxygraph (Oroboros Instruments GmbH, Innsbruck, Austria). Samples were resuspended in respiration buffer (0.5 mM EGTA, 3 mM MgCl_2_, 60 mM Lactobionic acid, 20 mM Taurine, 10 mM KH_2_PO_4_, 20 mM HEPES, 110 mM d-Sucrose, 1% fat-free BSA), and oxygen consumption rates [pmol/(s*mg synaptosomal proteins)] were measured at 37°C according to a substrate-uncoupler-inhibitor-titration (SUIT) protocol as previously described (Awadhpersad and Jackson, 2021).

Briefly, measurements were performed in the presence of 1 mM malate, 5 mM pyruvate and 5 mM glutamate, and additional substrates and inhibitors were injected to oxygraph chambers in the following order: (i) 1.25 mM ADP; (ii) 10 mM succinate; (iii) 1 μg/ml oligomycin A; (iv) titration of carbonyl cyanide 4-(trifluoromethoxy) phenylhydrazone (FCCP); (v) 0.5 μM rotenone; (vi) 1 μg/ml antimycin A; (vii) 2 mM ascorbate; (viii) 0.5 mM *N,N,N′,N′*-tetramethyl-*p*-phenylenediamine (TMPD); and (ix) 10 mM sodium azide. All substrates and inhibitors were purchased from Sigma Aldrich (Saint Louis, MO, United States). Polarographic oxygen sensors monitored changes in oxygen concentration as substrates and inhibitors were applied, and changes in oxygen concentration and consumption were plotted over time.

The DatLab software (Oroboros Instruments GmbH, Innsbruck, Austria) was used for calculating oxygen consumption rates (OCR). Rates were normalized to sample protein concentration, determined using the BCA protein assay kit (Pierce, Thermo Fisher Scientific) according to the manufacturer’s instructions.

CI and CII-linked coupled respiration was achieved after addition of succinate (ii), leak-respiration after uncoupling the ATP-synthase (CV) from the electron transport system using oligomycin, and maximal uncoupled respiration by titrating FCCP until no further increase in respiration was detected. Complex IV (CIV) -dependent respiration was achieved by addition of ascorbate (vii), TMPD (viii), and sodium azide (ix). The difference between oxygen consumption before and after addition of sodium azide was considered as CIV-dependent respiration. Residual oxygen consumption (ROX) values were achieved after addition of rotenone (v) and antimycin A (vi), and subtracted from all oxygen consumption rates to correct for non-mitochondrial oxygen consumption. The coupling control ratio (CCR) was calculated according to the protocol by the MitoEAGLE Task Group (Gnaiger et al., 2020).

### 2.6. Immunoblot analysis

#### 2.6.1. Sample preparation

For immunoblot analysis, synaptosome samples were diluted in lysis buffer (50 mM This-HCl pH 7.5, 100 mM NaCl, 1 mM DTT) with 1% n-Dodecyl-beta-Maltoside (DDM) (Thermo Fisher Scientific, Waltham, MA, United States), and protease and phosphatase inhibitors (Pierce Protease and Phosphatase Inhibitor Mini Tablets, Thermo Fisher Scientific), and incubated on ice for 15 min. Protein concentrations (μg/μl) were determined spectrophotometrically using the BCA protein assay kit (Pierce, Thermo Fisher Scientific) according to the manufacturer’s instructions.

#### 2.6.2. SDS-PAGE, electroblotting, and antibodies

Protein samples were mixed with Laemmli buffer containing β-mercaptoethanol, and proteins were separated in stain-free 4–20, 7.5%, or all kD pre-cast gels (TGX Stain-Free, BioRad, CA, United States) and electroblotted to PVDF membranes (Trans-blot Turbo Transfer pack, BioRad). Membranes were blocked in 5% milk/PBST for 60 min at r/t and incubated with primary antibodies against SYP (M0776, Dako; 1:500), HDAC2 (05–814, Millipore, 1:500), PSD-95 (610,495, BD Transduction Laboratories, 1:500), CDK-5 (05–364, Upstate, 1:2000), VDAC-1 (ab14734, Abcam; 1:4000), Vinculin (ab129002, Abcam; 1:4000), SDHA (ab14715, Abcam; 1:10000), mtCO1 (ab14705, Abcam; 1:3000), Atp5b (17247-1-AP, Proteintech, 1:5000), TOM40 (sc-11,414, Santa Cruz; 1:2000), TOM20 (11802-1-AP, Proteintech, 1:4000), OPA1 (612,606, BD Biosciences, 1,1,000), and β-tubulin (T4026, Sigma Aldrich; 1:5000) o/n at +8°C. Secondary antibodies against mouse (P0447, DAKO) and rabbit (P0399, DAKO), diluted 1:5000 in 1% milk/PBST +0.01% SDS, were incubated for 60 min at r/t. Antibody detection and signal intensity quantification was performed using the ChemiDoc XRS+ imaging system (BioRad, CA, United States) utilizing stain-free technology for total protein normalization, or the Odyssey Infrared Imaging system (LI-COR Biosciences).

### 2.7. Mitochondrial DNA copy number analysis

#### 2.7.1. Standard curve preparation

Mitochondrial DNA (mtDNA) copy number (cn) was quantified from cerebellar synaptosomes of P30 and P45 *Cstb^−/−^* and wild type mice using a standard curve-based method, as previously described (Rooney et al., 2015). Briefly, a standard curve of template copies using a plasmid [501–1 mtDNA in the pBR325 backbone (Blanc et al., 1981)] was amplified in a 2-fold dilution series ranging from 4,000 to 256,000 copies. The standard line was plotted as log (plasmid copy number) versus real-time quantitative PCR (RT-qPCR) cycle threshold (Ct). Ct values were obtained from the RT-qPCR amplification plots.

#### 2.7.2. DNA isolation from synaptosomes

Total DNA was isolated from synaptosomes (*n* = 10–13/genotype/age group, males and females) using the DNeasy Blood & Tissue Kit (Qiagen, Venlo, Netherlands) according to the manufacturer’s instructions. Prior to lysis, samples were resuspended in ice-cold 1xPBS, and 50% of the sample volume was removed and used for determination of protein concentration using the BCA protein assay kit (Pierce, Thermo Fisher Scientific) according to the manufacturer’s instructions. Stock DNA (sDNA) elution volume was set to 20 μl and samples of working concentration DNA (wDNA) of approximately 0.1 ng/μl were diluted of these. The concentrations of sDNA and wDNA were assessed using the Qubit dsDNA HS Assay Kit (Thermo Fisher Scientific).

#### 2.7.3. Real-time quantitative PCR

For each reaction, 1 μl of DNA template (standard or wDNA) was mixed with iQ SYBR Green Supermix (BioRad) and 10 uM of forward (5′-AGGAGCCTGTTCTATAATCGATAAA-3′) and reverse (5′-GATGGCGGTATATAGGCTGAA-3′) primers. RT-qPCR reactions were run once in triplicate using the CFX96 Real-time PCR detection system (BioRad) and the CFX Maestro software under the following program: 7 min at 95°C, 10 s at 95°C, and 40 cycles of 30 s at 60°C, followed by a melt curve protocol of 0.5 s + plate read at 65°C, and then 0.5 s + plate read at each 0.5°C increments between 65°C and 95°C. MtDNA copy number per μl wDNA was determined using Ct-values of the unknown samples in relation to the standard curve, and further calculated to mtDNA copy number/mg protein. RT-qPCR assays were performed in accordance with MIQE guidelines (Bustin et al., 2009).

### 2.8. Electron microscopy

Cerebellar tissue pieces (*n* = 1–3; P30, P45) from *Cstb^−/−^* and wild type mice were fixed in 2% glutaraldehyde in phosphate buffer, post-fixed in 1% osmium tetroxide and dehydrated through ascending concentrations of alcohol and embedded in Epon 812 resin. 60 nm ultrathin sections were obtained on a Reichert-Jung Ultracut ultramicrotome (Leica Microsystems, Wetzlar, Germany) equipped with a Diatome diamond knife (Diatome Ltd., Nidau, Switzerland), transferred to copper grids, stained with uranyl acetate and lead citrate, and observed in a CM12 transmission electron microscope (Philips Healthcare, Amsterdam, the Netherlands) at 80 kV. Images were recorded with a Morada digital camera and analyzed using the iTEM software (ResAlta Research Technologies, Golden, CO, United States).

### 2.9. Statistical analyses

Statistical analyses were carried out using GraphPad Prism version 9.4 for Windows (GraphPad Software, La Jolla, CA, United States^3^). Data was tested for normal distribution and comparisons between experimental conditions were evaluated using the two-tailed unpaired *t*-test, the nonparametric Mann–Whitney *U* test, and the one-way ANOVA with the Šídák correction. Statistical significance was defined as *p* < 0.05.

## 3. Results

### 3.1. Differential abundance of mitochondrial and synaptic proteins in *Cstb*^−/−^ synaptosomes

To investigate synaptic alterations associated with the early symptomatic phase of CSTB deficiency, we used label-free quantitative proteomics to analyze cerebellar synaptosomes from P30 *Cstb^−/−^* and wild type mice. Principal component analysis (PCA) showed that the first component, PC1, explained 34.3% of the total variation, and segregated the samples into two genotype-specific clusters (Supplementary Figure 1A). We identified more than 2,500 and reliably quantified 1,555 proteins (Supplementary Table 1_sheet1). Of these, 349 proteins differed significantly between *Cstb^−/−^* and wild type mice (Supplementary Figure 1B and Supplementary Table 1_sheet2). In addition, we identified 34 proteins in one genotype only (Supplementary Table 1_sheet3).

**Figure 2:**
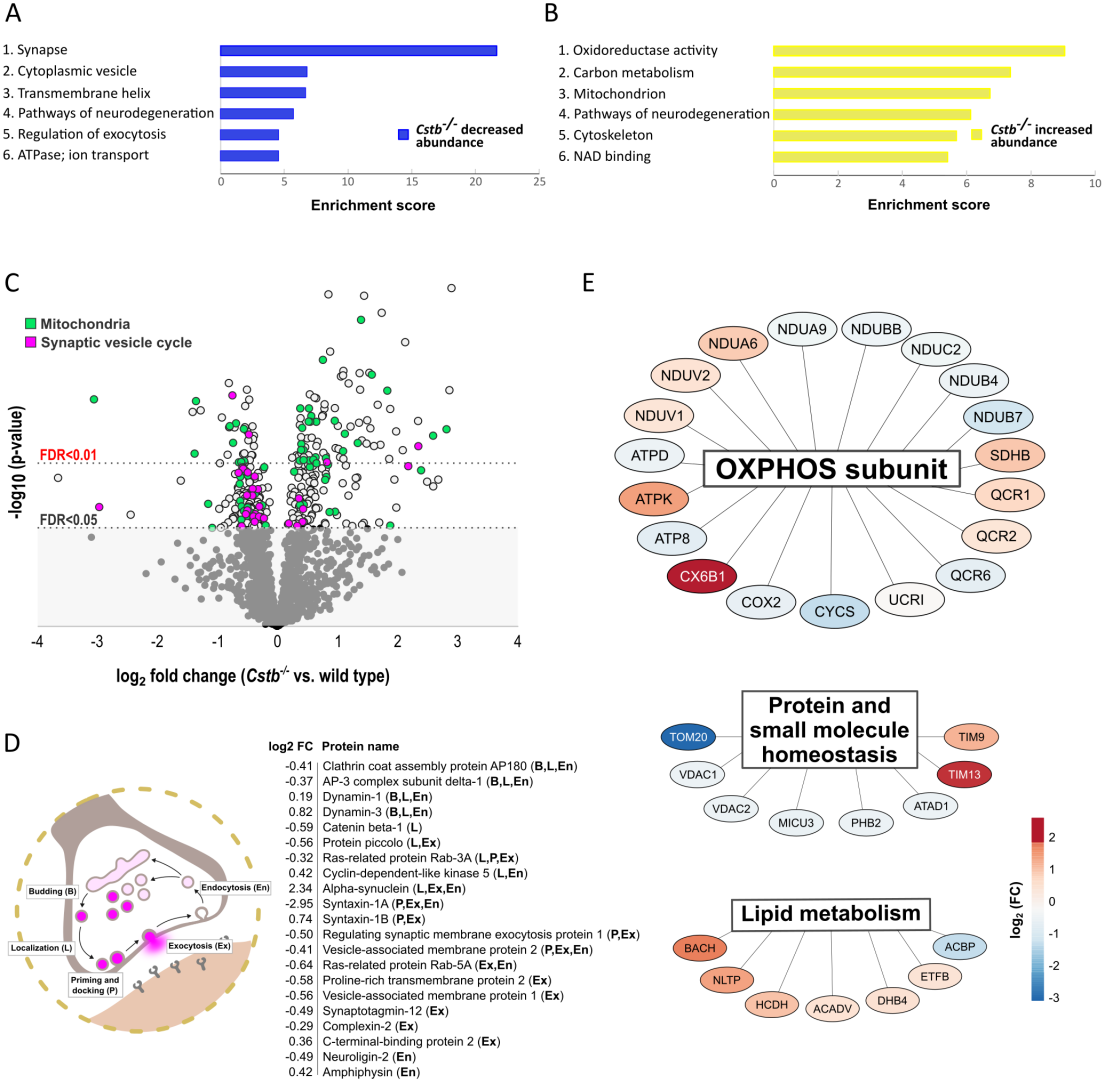
Differentially abundant proteins between wild type and *Cstb^−/−^* mice synaptosomes at P30. **(A,B)** Bar charts of enriched (enrichment score > 4.0) DAVID functional annotation clusters of proteins with decreased **(A)** and increased **(B)** abundance in *Cstb^−/−^* synaptosomes. **(C)** Volcano plot of differentially abundant proteins plotted as –log10-transformed value of *p* [derived from the *t*-test (*Cstb^−/−^*/wild type; *n* = 5 + 4)] vs. log2 fold change protein abundance. The 349 DAPs significantly differing in abundance (*q*-value ≤0.05) are plotted above the threshold limit of –log10 (value of *p*) 1.62. Mitochondrial proteins (*n* = 66) are plotted in green and proteins of the synaptic vesicle cycle (*n* = 30) in purple. The remaining quantified proteins (1206) with non-significant *q*-values are plotted in the gray area below the threshold limit. **(D)** Schematic figure of the synaptic vesicle cycle and the differentially abundant synaptic vesicle cycle proteins (*n* = 21) with fold changes (*Cstb^−/−^*/wt), labeled according to function (B, budding; L, localization; P, priming and docking; Ex, exocytosis; En, endocytosis). **(E)** A subset of the differentially abundant mitochondrial proteins grouped by biological function. Most proteins (*n* = 19) are subunits of the oxidative phosphorylation (OXPHOS), followed by proteins involved in homeostasis of proteins and small molecules (*n* = 8) and lipid metabolism (*n* = 7). Red and blue color indicate increased and decreased protein abundance (*Cstb^−/−^*/wild type), respectively.

To investigate the biological relationships between the 349 differentially abundant proteins, we performed Gene Ontology (GO) classification (Ashburner et al., 2000; Gene Ontology Consortium, 2021), DAVID functional annotation clustering (Huang et al., 2009a,b), Reactome pathway analysis (Wu and Haw, 2017), and PANTHER enrichment analysis (Mi et al., 2013). Analysis of statistically significant (FDR <0.01) overrepresented GO terms revealed that Synapse and Mitochondria were the most overrepresented cellular components in the dataset (Supplementary Figure 1B and Supplementary Table 2_sheet1). Correspondingly, the top 15 GO terms of biological processes were associated with energy metabolism and nucleotide biosynthesis and they grouped into several overlapping GO-terms (Supplementary Figure 2 and Supplementary Table 2_sheet2). The DAVID functional annotation clustering tool annotated proteins with increased abundance (*n* = 188) to mitochondrial function and energy metabolism, whereas proteins with decreased abundance (*n* = 161) were annotated to synaptic structure and function (Figures 2A,B, Supplementary Table 2_sheet3, and Supplementary Table 2_sheet4). The Reactome pathway database annotated the differentially abundant proteins to pathways related to immunological functions, mitochondrial energy metabolism, and intracellular trafficking (Supplementary Figure 3 and Supplementary Table 2_sheet5).

### 3.2. Alterations in proteins of the synaptic vesicle cycle and mitochondrial metabolism in *Cstb*^−/−^ synaptosomes

Grouping of the differentially abundant proteins by their biological mechanisms revealed that a substantial number were members of the synaptic vesicle cycle or functioned within the mitochondria (Figure 2C). Of the synaptic vesicle cycle proteins, 21 were direct members and an additional 9 were regulators of this pathway (Supplementary Table 3_sheet1). A majority of these were decreased in *Cstb^−/−^* synaptosomes. Most of these altered proteins function in localizing synaptic vesicles to the active zone, the fusion and release of neurotransmitters, and the retrieval of synaptic vesicle proteins by endocytosis (Figure 2D).

Of all significantly different proteins, 66 were mitochondrial (Figure 2C and Supplementary Table 3_sheet2). Of these, 28 were decreased and 38 increased in abundance. The single largest group was oxidative phosphorylation (OXPHOS) subunits, which were distributed across complexes I–V (Figure 2E). The highest fold changes were observed for complex I, complex IV and complex V subunits, with abundance of complex I subunits being decreased and complex IV and complex V subunits increased in *Cstb^−/−^* synaptosomes. Altered abundance was also observed for components of the TOM and TIM mitochondrial import translocon complexes and several other proteins involved in protein and small molecule homeostasis (Figure 2E). In addition, the proteome analysis showed changes in proteins involved in lipid metabolism, including acyl-coenzyme A hydrolysis and lipid transport (Figure 2E).

**Figure 3:**
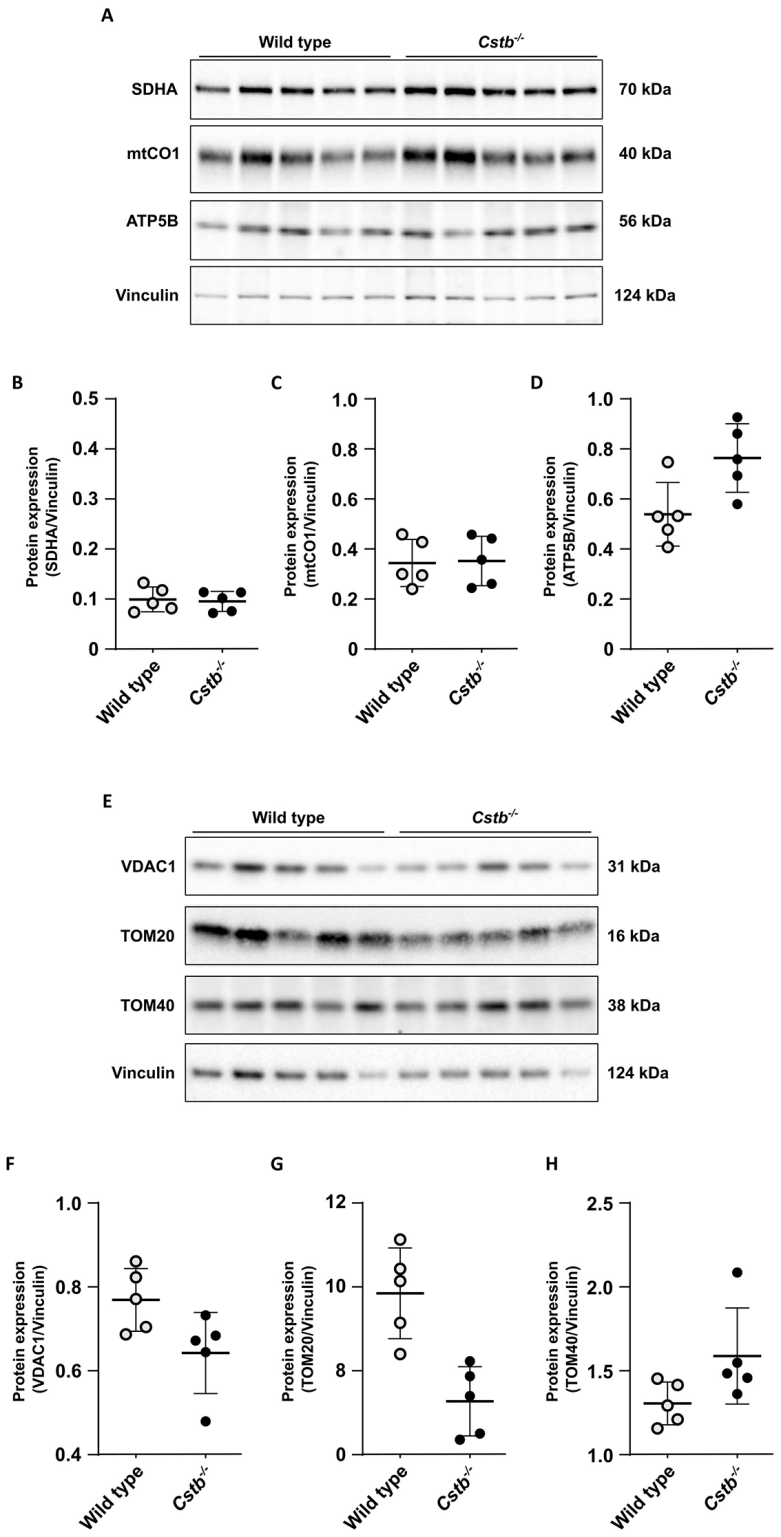
Immunoblot detection of mitochondrial proteins in wild type and *Cstb^−/−^* cerebellar synaptosomes at P30. Representative immunoblot detection **(A,E)** and relative expression of SDHA **(B)**, mtCO1 **(C)**, ATP5B **(D),** VDAC1 **(F)**, TOM20 **(G)**, and TOM40 **(H)** in synaptosomes of wild type (white) and *Cstb^−/−^* (black) mice (*n* = 5 + 5). Antibody intensity values were normalized to that of Vinculin. Bars represent mean, and error bars standard deviation (SD).

We further examined differences in the mitochondrial proteins by immunoblot analysis of individual OXPHOS complexes and mitochondrial outer membrane proteins (Figures 3A,E). We did not detect clear genotype-specific changes in the expression of marker proteins for OXPHOS complexes II (succinate dehydrogenase flavoprotein subunit; SDHA), IV (cytochrome c oxidase subunit 1; mtCO1), and V (ATP synthase subunit beta; ATP5B) (Figures 3B–D), neither in the expression of Voltage-dependent anion-selective channel protein 1 (VDAC1), Mitochondrial import receptor subunit TOM20 homolog (Tom20), nor Mitochondrial import receptor subunit TOM40 homolog (Tom40), although variation was high within genotypes (Figures 3F–H).

### 3.3. Mitochondrial respiration declines progressively in *Cstb*^−/−^ synaptosomes

To test whether alterations to the mitochondrial proteome in *Cstb^−/−^* synaptosomes affected organelle function, we performed high-resolution respirometry to investigate oxidative phosphorylation in synaptosomal preparations. We analyzed mice at the early-symptomatic stage (P30) and later once there are detectable myoclonus phenotypes and signs of neuronal loss (P45). These two time points allowed us to determine if any alterations in mitochondrial oxidative phosphorylation function preceded the onset of the clinical symptoms in *Cstb^−/−^* mice.

**Figure 4:**
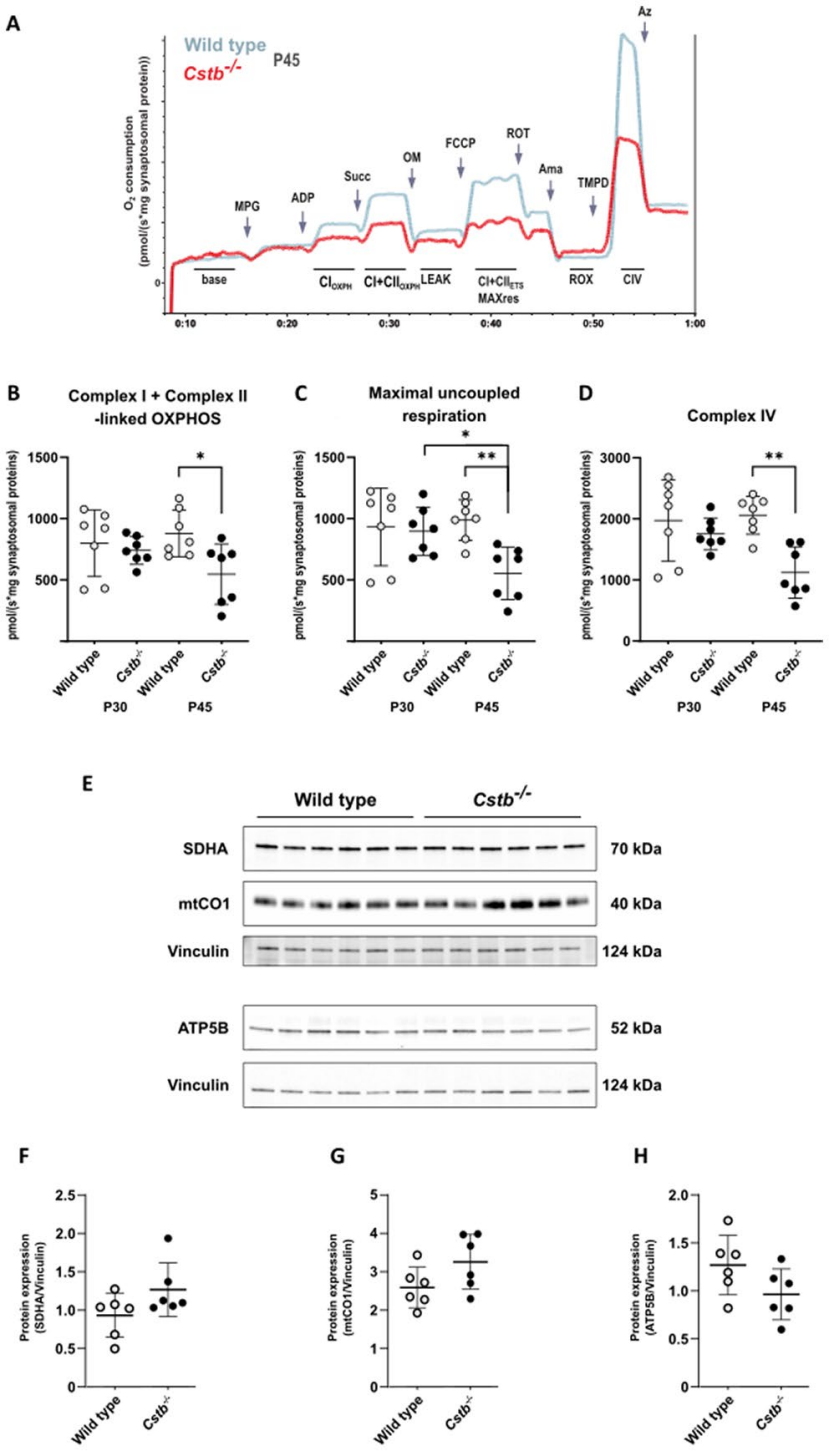
Mitochondrial respiration in wild type and *Cstb^−/−^* synaptosomes. **(A)** Representative traces of the SUIT-protocol from wild type and *Cstb^−/−^* samples at P45 showing oxygen consumption consumption (pmol/(s*mg synaptosomal protein); y-axis) during substrate and coupling states (x-axis). MPG, malate/pyruvate/glutamate; ADP, adenosine diphosphate; Succ, succinate; OM, oligomycin; FCCP, carbonyl cyanide-*p*-trifluoromethoxyphenylhydrazone; ROT, rotenone; Ama, antimycin A; TMPD, *N,N,N,N*-tetramethyl-*p*-phenylenediamine; Az, azide; CI_OXPH_, phosphorylating respiration (OXPHOS) in presence of CI substrates; CI + CII_OXPH_, OXPHOS respiration in presence of CI and CII substrates; LEAK, oligomycin-inhibited non-phosphorylating basal respiration; CI + CII_ETS_ MAX_res_, maximal capacity of the electron transport system; ROX, residual oxygen consumption; CIV, complex IV activity. **(B–D)** Complex I + Complex II-linked OXPHOS **(B)**, Maximal uncoupled respiration **(C)**, and Complex IV activity **(D)** in wild type (gray) and *Cstb^−/−^* (black) synaptosomes at P30 and P45 (*n* = 7 + 7 + 7 + 7). Statistical significance was determined by one-way ANOVA with correction for multiple comparisons using the Šídák method; *p < 0.05; **p < 0.01; bars represent mean and error bars standard deviation of oxygen consumption [pmol/(second*mg synaptosomal protein)]. **(E)** Representative immunoblot detection and relative expression of SDHA **(F)**, mtCO1 **(G)**, and ATP5B **(H)** in synaptosomes of wild type (white) and *Cstb^−/−^* (black) mice (*n* = 6 + 6) at P45. Antibody intensity values were normalized to that of Vinculin. Bars represent mean, and error bars standard deviation (SD).

Following synaptosome isolation, samples were immediately recorded for mitochondrial respiration. Associated with CSTB deficiency we observed a progressive decline in oxidative phosphorylation capacity. At P30, we found no differences between genotypes in any of the examined respiration states, but at P45 the overall respiration declined significantly in *Cstb^−/−^* mice (Figure 4A). ADP-linked complex I and combined succinate-induced complex I and complex II oxygen-consumption rates were all reduced in *Cstb^−/−^* mice, implying for decreased utilization of complex I and complex II substrates. Both coupled (Figure 4A) and uncoupled (Figure 4C) respiration was reduced in *Cstb^−/−^* mice, implying that the respiratory dysfunction is not dependent on complex V. Oligomycin-induced leak respiration, indicative of damaged mitochondrial inner membrane, was lower in *Cstb^−/−^* samples, but the difference was caused by increased leak respiration in wild type mice (Supplementary Figure 4). Such increase was not observed in *Cstb^−/−^* mice. The coupling control ratio showed no differences in synaptosome samples between genotypes at either time point (Supplementary Figure 5). Independent complex IV activity was also reduced in *Cstb^−/−^* mice (Figure 4D), further pointing toward an oxidative phosphorylation complex impairment and general respiratory dysfunction. Immunoblot analysis for nuclear and mitochondrial encoded subunits of the OXPHOS complexes showed no difference in electron transport system proteins, complex II and complex IV subunits SDHA and mtCO1, or in the complex V subunit, ATP5B at P45 (Figures 4E–H).

### 3.4. No change in mitochondrial DNA copy number and membrane ultrastructure in *Cstb*^−/−^ cerebella

**Figure 5:**
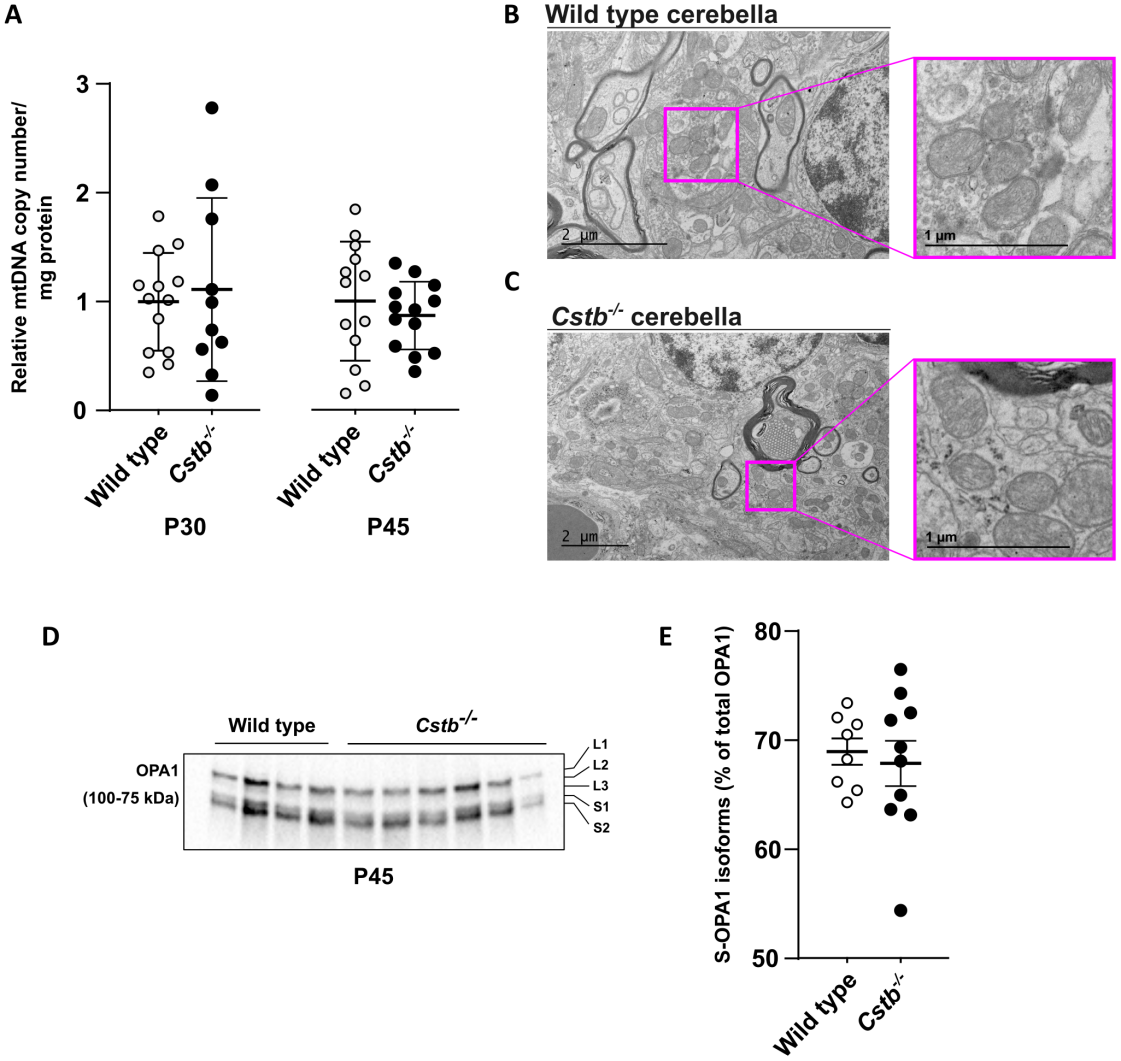
Mitochondrial DNA copy number and membrane ultrastructure is not altered in *Cstb^−/−^* cerebella. **(A)** The relative ratio between mtDNA copy number and protein amount in synaptosomes of wild type (white) and *Cstb^−/−^* (black) mice cerebella at P30 (*n* = 10–13) and P45 (*n* = 13). Bars represent mean, and error bars standard deviation (SD) of relative mtDNA copy number/mg synaptosomal protein. **(B,C)** Transmission electron microscopy images showing mitochondrial ultrastructure in wild type **(B)** and *Cstb^−/−^* **(C)** cerebellar tissue at P45. **(D)** Representative immunoblot detection using an antibody against OPA1 in synaptosomes of wild type and *Cstb^−/−^* mice (*n* = 4 + 6) at P45. The long and short OPA1 isoforms are marked L1-L3 and S1-S2, respectively. **(E)** The short isoforms of OPA1 (S-OPA1) in relation to total OPA1 in synaptosomes of wild type (white) and *Cstb^−/−^* (black) mice at P45 (*n* = 8–10). Bars represent mean, and error bars standard deviation (SD).

Next, we investigated whether other mitochondrial phenotypes were affected in *Cstb^−/−^* cerebella. No significant differences in mitochondrial DNA (mtDNA) copy number were detected in synaptosomal preparations between *Cstb^−/−^* and wild type mice at P30 and P45 (Figure 5A). Further, no gross alterations in the mitochondrial membrane ultrastructure were observed in cerebellar tissue preparations using transmission electron microscopy (TEM) (Figures 5B,C). We also investigated early indicators of cellular stress affecting mitochondrial dynamics by analyzing the (OPA1) isoforms in synaptosomal preparations. The proteolytic cleavage of the long isoforms (L-OPA1) was estimated as a percentage of short isoforms (S-OPA1) of total OPA1. No differences were observed between *Cstb^−/−^* and wild type mice (Figures 5D,E).

## 4. Discussion

Identifying the mechanisms underlying the synaptic pathophysiology associated with CSTB deficiency is important for understanding EPM1 disease onset and progression. In mice, CSTB deficiency causes progressive neuron loss, which is most striking in the cerebellum, the emergence of which coincides with the onset of myoclonus (Pennacchio et al., 1998) and is preceded by prevalent rearrangements of synaptic proteins in the cerebellum (Gorski et al., 2020) and altered GABAergic signaling in cerebellar Purkinje cells (Joensuu et al., 2014). Following our previous study from presymptomatic *Cstb^−/−^* mice (Gorski et al., 2020), we here extended the analysis of cerebellar synaptosomes to the early symptomatic phase. We identified an impairment in mitochondrial respiration, which was preceded by widespread changes in the mitochondrial proteome. Our data reveal that mitochondrial dysfunction contributes to the early pathogenesis of CSTB deficiency.

Neurons are metabolically active and have a high energy demand, most of which is used for neurotransmission (Harris et al., 2012). Synaptic energy in the form of adenosine triphosphate is synthetized locally through glycolysis and mitochondrial oxidative phosphorylation (OXPHOS) (Harris et al., 2012). Presynaptic mitochondrial dysfunction and bioenergetic failure have been associated with several, both common and rare neurodegenerative diseases and their models (reviewed in Li and Sheng, 2022), and our findings presented here establish the presence of EPM1 among these disorders. However, the question remains how deficiency of a protein with a cytosolic and nuclear localization can cause failure of synaptic mitochondria without evident alterations in the mitochondrial phenotype.

Cystatin B is a soluble cytoplasmic protein that associates with cytoplasmic granular structures representing lysosomes (Alakurtti et al., 2005). It has previously been reported to have synaptic localization with an implied crucial role in synaptic physiology (Penna et al., 2019; Gorski et al., 2020). CSTB belongs to the cystatin superfamily of endogenous inhibitors of lysosomal cysteine proteases of the cathepsin family that are thought to protect cells from cathepsin-mediated proteolysis in the cytoplasm in the case of lysosomal membrane damage (Boya and Kroemer, 2008). Leakage of lysosomal cathepsins has been reported in several pathological conditions and neurodegenerative disorders (Nagai et al., 2000; Sundelöf et al., 2010; Morena et al., 2017), and mutations in mitochondrial genes are often associated with impairment of the lysosomal system and *vice versa* (Deus et al., 2020). Increased activity of cathepsin B, one of the cysteine proteases inhibited by CSTB, has been described in lymphoblastoid cells from EPM1 patients (Rinne et al., 2002), in cultured cerebellar granule neurons from *Cstb^−/−^* mice (Lehtinen et al., 2009), and in neural progenitor cells from *Cstb^−/−^* mice (Daura et al., 2021). Cathepsin B maintains proteolytic activity in the cytoplasm and initiates mitochondrial apoptosis though activation of Bcl-2 family members (Droga-Mazovec et al., 2008; de Castro et al., 2016). Indeed, apoptotic cell death of cerebellar granule neurons is one of the hallmarks of brain pathology in *Cstb^−/−^* mice (Pennacchio et al., 1998) with cystatin B-cathepsin B double knockout mice showing a reduction in the amount of cerebellar granule neuron apoptosis compared to *Cstb^−/−^* mice (Houseweart et al., 2003). Recently, lysosomal leakage and consequent increased proteolytic activity of cathepsins B and L were reported to initiate a metabolic remodeling of the mitochondrial proteome in a human iPSC-derived macrophage model (Bussi et al., 2022). This lysosome-mitochondria crosstalk was implied to modulate macrophage immunometabolism *via* cathepsin-mediated degradation of mitochondrial proteins leading to impaired OXPHOS activity (Bussi et al., 2022). It remains to be investigated whether similar mechanisms contribute to compromised mitochondrial function in neurons. Interestingly, a more than twofold increase in cathepsin B abundance was observed in our proteomics data of cerebellar synaptosomes from *Cstb^−/−^* mice. Considering that cathepsin B also regulates lysosome and autophagosome dynamics (Man and Kanneganti, 2016; Qi et al., 2016), and activates the NLRP3 neuroinflammasome (Chevriaux et al., 2020) and subsequent production of interleukin (IL) 1β (Bai et al., 2018), all of which are implicated in experimental models of CSTB deficiency (Maher et al., 2014; Polajnar et al., 2014), it is possible that many of the pathological consequences of CSTB deficiency, including mitochondrial dysfunction, are mediated through increased cathepsin B activity.

The polarized structure and compartmentalized functions of neurons require long-distance transport of a variety of cargoes, including organelles and synaptic vesicle precursors (Maday et al., 2014). In addition to housing metabolic pathways, mitochondria form contact sites with other organelles to modulate the exchange of lipids, ions, and proteins (Vance, 2014). Cellular stress has been shown to increase contact sites between the endoplasmic reticulum (ER) and mitochondria *in vitro* (Bravo et al., 2011), and for several neurodegenerative disorders, including Alzheimer’s disease, Parkinson’s disease, and Charcot–Marie–Tooth disease, many dysregulated cellular functions have been associated with these (Wilson and Metzakopian, 2021). Impaired mitochondrial respiration in *Cstb^−/−^* synaptosomes could result from reduced or dysregulated mitochondria-ER signaling, causing destabilization of OXPHOS supercomplexes. This has been reported in a neuronal model of Alzheimer’s disease, where loss of mitochondria-ER contact sites leads to dysfunctions in mitochondrial bioenergetics due to reduced levels of cardiolipin, a phospholipid that stabilizes OXPHOS supercomplexes in the mitochondrial inner membrane (Martino Adami et al., 2019).

Impaired redox homeostasis has previously been implicated as a key mechanism by which CSTB deficiency causes neuronal death and oxidative damage in the cerebellum of *Cstb^−/−^* mice (Lehtinen et al., 2009). In addition, mitochondrial dysfunction has been demonstrated in several *in vitro* models derived from *Cstb^−/−^* mice, leading to propositions of both direct and indirect mechanisms linking CSTB function to mitochondria. Maher et al. (2014) investigated inflammatory responses in lipopolysaccharide (LPS)-stimulated bone marrow-derived macrophages from *Cstb^−/−^* mice and showed that mitochondrial membrane potential stability is impaired upon LPS stimulation and leads to increased ROS generation (Maher et al., 2014). The authors suggested that CSTB translocates to mitochondria where it physically protects the mitochondrial membrane integrity (Maher et al., 2014). More recently, in a cell culture model of murine neural stem cell renewal and differentiation, activation of nuclear-encoded mitochondrial genes was shown to be delayed in CSTB-deficient cells leading to impairment of the enhanced mitochondrial respiration that is induced upon induction of differentiation (Daura et al., 2021). We previously reported proteomic alterations in cerebellar synaptosomes of presymptomatic *Cstb^−/−^* mice and showed extensive rearrangements of the mitochondrial proteome, especially in proteins involved in mitochondrial energy metabolism, ROS production, and antioxidant-mediated maintenance of redox homeostasis (Gorski et al., 2020). Since the data also showed changes in several key structural transport proteins, previously reported on gene expression level (Joensuu et al., 2014), we suggested that defects in axonal transport could contribute to synaptic mitochondrial dysfunction (Gorski et al., 2020). None of these studies reported alterations in mitochondrial morphology. Alterations in mitochondrial membrane morphology are often associated with organelle dysfunction (Bertholet et al., 2016). In the present study, we did not identify alterations in the abundance of key factors that coordinate mitochondrial membrane fission and fusion. Furthermore, cleavage of the long isoform of OPA1, which regulates mitochondrial cristae integrity, mtDNA maintenance, and mitochondrial inner membrane fusion, was not altered in the synaptic mitochondria from *Cstb^−/−^* mice. Taken together, these data suggest that mitochondrial dysfunction in CSTB deficiency is due to a secondary event rather than primary defect to organelle function.

It is likely that the consequences of reduced mitochondrial respiration affect the energy-demanding downstream functions of the synapse, including neurotransmission. Synaptic vesicle refilling and recycling are processes sensitive for energy depletion (Pathak et al., 2015). Indeed, in the present proteomics data, we observed differential abundance of several members involved in the synaptic vesicle cycle, predicted to affect synaptic vesicle mobilization, docking and fusion (John et al., 2021). The previously implicated alterations in GABAergic inhibition in brains of *Cstb^−/−^* mice (Joensuu et al., 2014) may thus be, at least partially, a consequence of impaired synaptic mitochondrial function. Whether the mitochondrial dysfunction is exclusive to cerebellar synaptosomes or applies also to other cell compartments or cell types, needs to be clarified in future studies.

In conclusion, our study shows that significant alterations in the synaptic proteome and consequent mitochondrial dysfunction are early changes in cerebellar synaptosomes of *Cstb^−/−^* mice, coinciding with the onset and progression of clinical symptoms. Understanding the underlying mechanisms is a prerequisite for designing new therapeutic strategies for EPM1.

## Data availability statement

The mass spectrometry proteomics data have been deposited to the ProteomeXchange Consortium *via* the PRIDE partner repository with the dataset identifier PXD040382.

## Ethics statement

The animal study was reviewed and approved by The Animal Ethics Committee of the State Provincial Office of Southern Finland (decisions ESAVI/10765/2015 and ESAVI/471/2019).

## Author contributions

KG, CJ, BB, and A-EL contributed to the study design. KG, CJ, TN, and VR performed the experiments. KG, CJ, TN, and BB analyzed the data. KG, BB, and A-EL wrote the manuscript. All authors discussed and commented on the manuscript.

## Funding

This work was supported by Folkhälsan Research Foundation (A-EL), the Sigrid Jusélius Foundation (A-EL), Medicinska Understödsföreningen Liv och Hälsa r.f. (A-EL), the Sigrid Juselius Foundation Senior Investigator Award (BB), Academy of Finland Research Fellowship (CJ), and the Magnus Ehrnroot Foundation (CJ). Mass spectrometry-based proteomic analyses were performed by the Proteomics Core Facility, Department of Immunology, University of Oslo/Oslo University Hospital, which is supported by the Core Facilities program of the South-Eastern Norway Regional Health Authority. This core facility is also a member of the National Network of Advanced Proteomics Infrastructure (NAPI), which is funded by the Research Council of Norway INFRASTRUKTUR-program (project number: 295910).

## Conflict of interest

The authors declare that the research was conducted in the absence of any commercial or financial relationships that could be construed as a potential conflict of interest.

## Publisher’s note

All claims expressed in this article are solely those of the authors and do not necessarily represent those of their affiliated organizations, or those of the publisher, the editors and the reviewers. Any product that may be evaluated in this article, or claim that may be made by its manufacturer, is not guaranteed or endorsed by the publisher.

## Footnotes

^1^ http://proteomecentral.proteomexchange.org

^2^ https://inkscape.org

^3^ www.graphpad.com
