# Supplementary material for: Progressive mitochondrial dysfunction in cerebellar synaptosomes of cystatin B-deficient mice
Source: Front Mol Neurosci. 2023 May 12;16:1175851. doi: 10.3389/fnmol.2023.1175851 (PMC10213208; doi:10.3389/fnmol.2023.1175851)
Supplement: Supplementary file 4 [file Data_Sheet_1.PDF]

Supplementary Material

Progressive mitochondrial dysfunction in cerebellar synaptosomes of cystatin B-deficient mice

Katarin Gorski [1,2], Christopher B. Jackson [3], Tuula A. Nyman [4], Veronika Rezov [1,2], Brendan J. Battersby [5], Anna-Elina Lehesjoki [1,2*]

*** Correspondence:** Anna-Elina Lehesjoki: anna-elina.lehesjoki@helsinki.fi

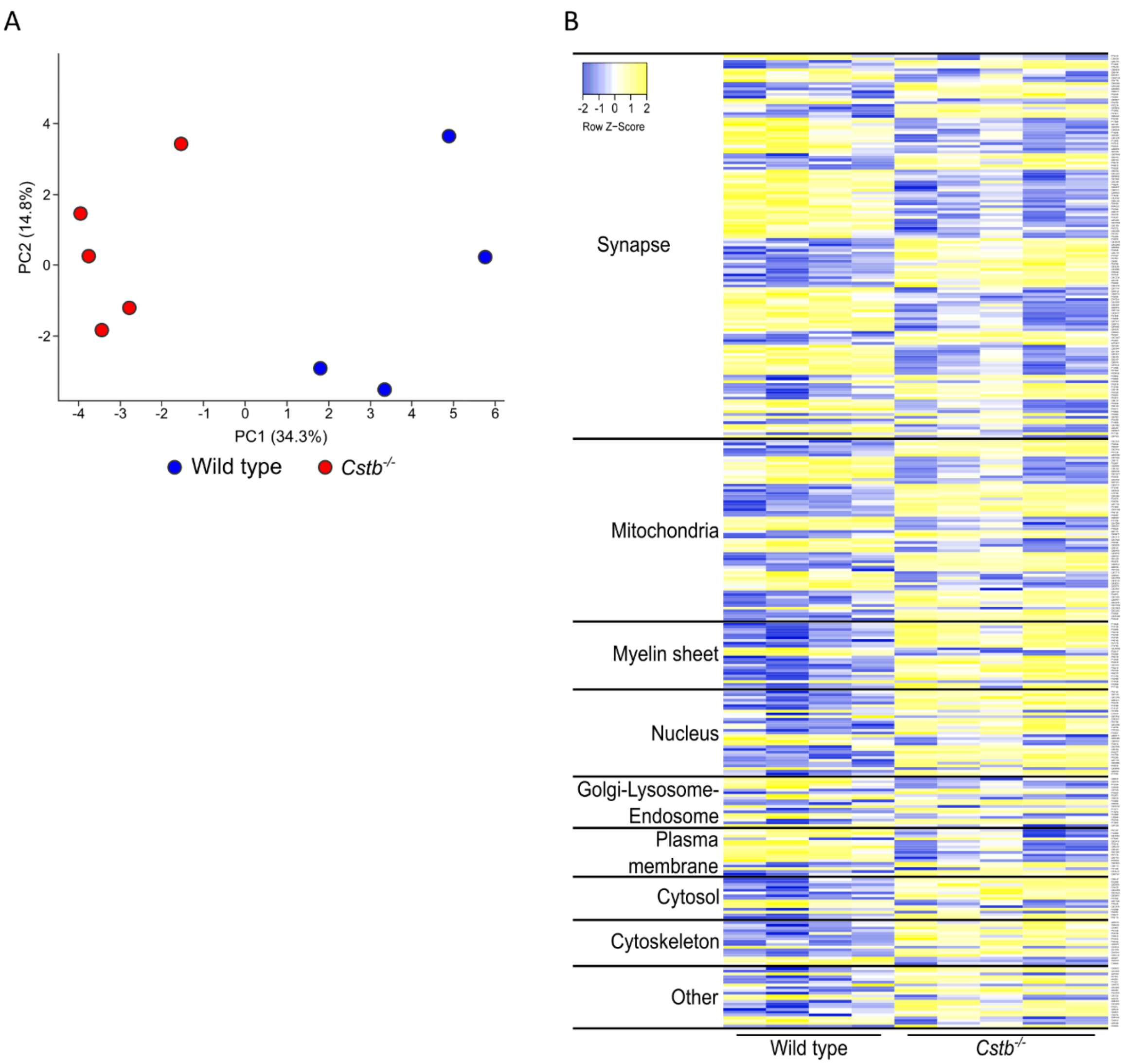

Supplementary figure 1. Differentially abundant cerebellar proteins between wild type and *Cstb*$^{-/-}$ synaptosomes at P30. (A) PCA plot of the proteomics data from cerebellar synaptosomes. Each dot represents one biological replicate, color coded for genotype. Blue and red data points represent wild type and *Cstb*$^{-/-}$ synaptosomes, respectively. **(B)** Heatmap of the 349 differentially abundant proteins, grouped according to Gene Ontology annotations for Cellular component with Uniprot ID:s shown on the right. Mitochondrial proteins were manually retrieved from Mitocarta3.0. Yellow and blue colors indicate increased and decreased abundance, respectively. Each column represents an independent sample, and each row represents a protein.

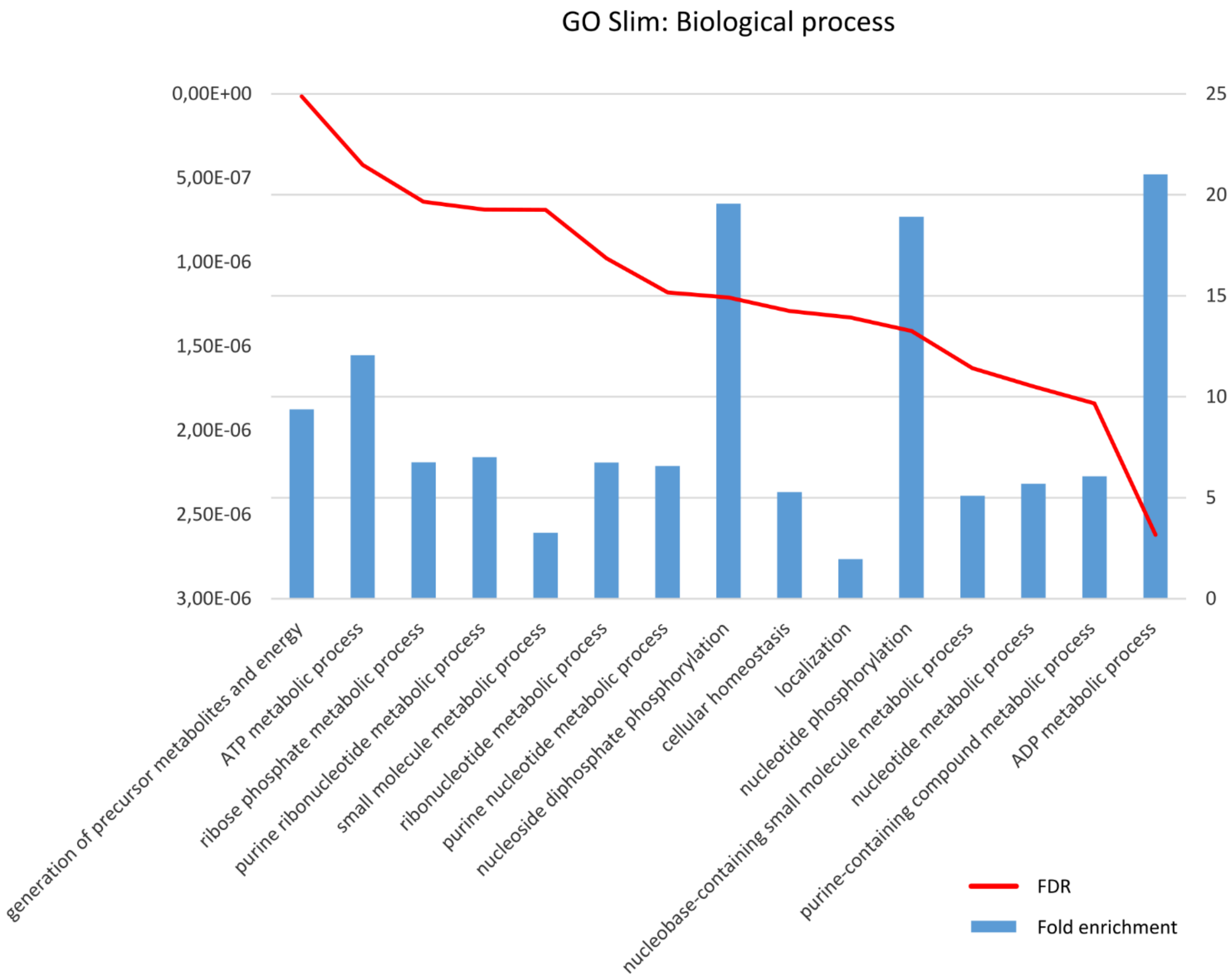

Supplementary figure 2. Gene Ontology (GO) analysis of 349 differentially abundant proteins in *Cstb*$^{-/-}$ synaptosomes. Bar chart of top 15 enriched (FDR<0.05) GO-terms show a high number of energy metabolism and nucleotide biosynthesis –related biological processes in the DAPs dataset. Fold enrichment (expected/observed) and FDR for each GO-term are marked on the x- and y-axes, respectively.

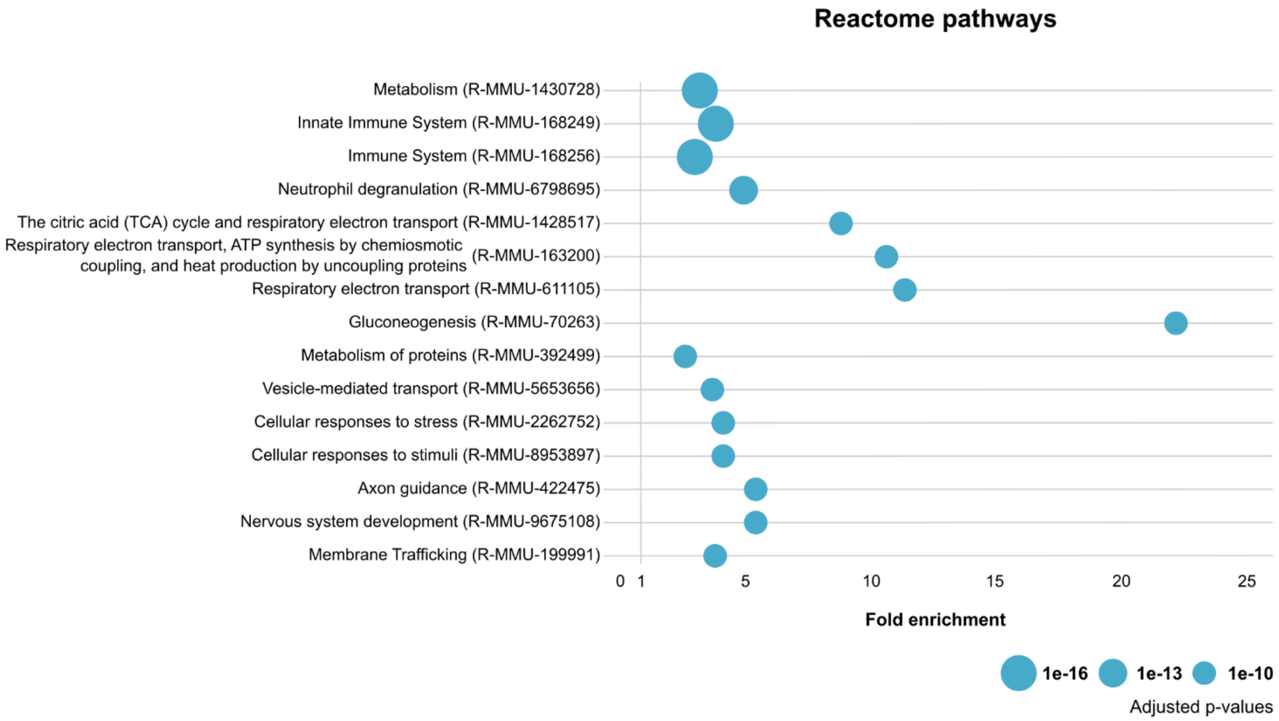

Supplementary figure 3. Reactome analysis of 349 differentially abundant proteins in *Cstb*$^{-/-}$ synaptosomes. Dot plot of top 15 enriched (FDR<0.05) Reactome pathways show an enrichment of immune system, energy metabolism, homeostasis, and transport –related terms in the DAPs dataset. Each circle represents a Reactome pathway term, circle size corresponding to the FDR of the term, and circle placement on the x-axis corresponding to its fold enrichment (expected/observed). The threshold limit of 1 (no fold change) is plotted as a grey vertical line.

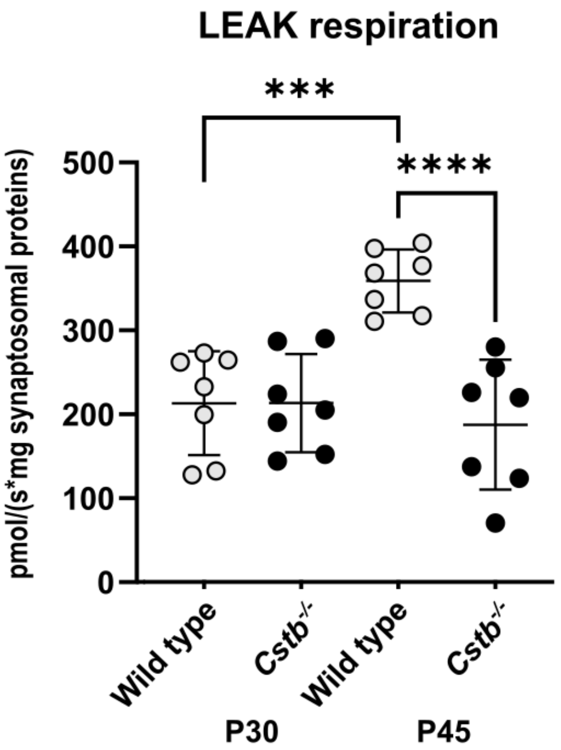

Supplementary figure 4. Leak respiration in *Cstb*$^{-/-}$ and wild type cerebellar synaptosomes. Oligomycin-induced leak-respiration in wild type (grey) and *Cstb*$^{-/-}$ (black) synaptosomes at P30 and P45. (n=7+7+7+7). Statistical significance was determined by one-way ANOVA with correction for multiple comparisons using the Šídák method; *** $p < 0.001$; **** $p < 0.0001$; bars represent mean and error bars standard deviation of oxygen consumption (pmol/(second*mg synaptosomal protein input)).

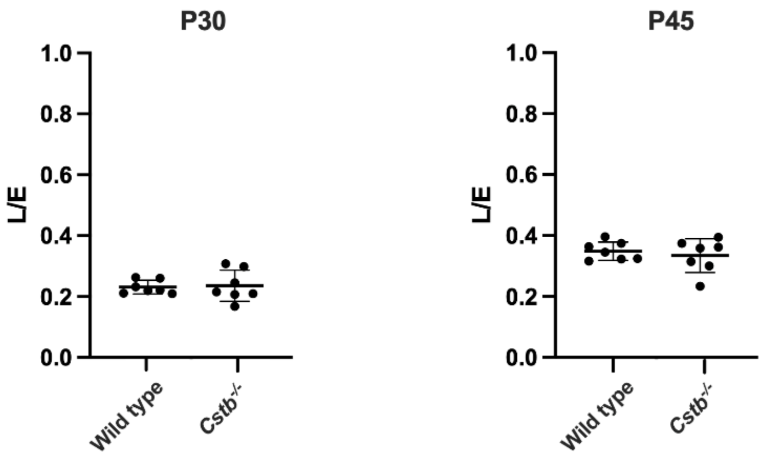

Supplementary figure 5. Coupling control ratio. Each dot represents a synaptosome preparation from an independent mouse with the indicated genotype. (L – leak, E – electron transfer capacity), Mean +/- SD.
